# Leukocytoclastic vasculitis in patients with IL12B or IL12RB1 deficiency: case report and review of the literature

**DOI:** 10.1186/s12969-021-00623-0

**Published:** 2021-08-13

**Authors:** Niusha Sharifinejad, Seyed Alireza Mahdaviani, Mahnaz Jamee, Zahra Daneshmandi, Afshin Moniri, Majid Marjani, Payam Tabarsi, Parisa Farnia, Mahsa Rekabi, Mazdak Fallahi, Seyedeh Atefeh Hashemimoghaddam, Masoumeh Mohkam, Jacinta Bustamante, Jean-Laurent Casanova, Davood Mansouri, Ali Akbar Velayati

**Affiliations:** 1 Non-Communicable Diseases Research Center, Alborz University of Medical Sciences, Karaj, Iran; 2 Pediatric Respiratory Diseases Research Center, National Research Institute of Tuberculosis and Lung Diseases (NRITLD), Shahid Beheshti University of Medical Sciences, Tehran, Iran; 3 Pediatric Nephrology Research Center, Research Institute for Children’s Health, Shahid Beheshti University of Medical Sciences, Tehran, Iran; 4 Pediatric Infections Research Center, Research Institute for Children’s Health, Shahid Beheshti University of Medical Sciences, Tehran, Iran; 5 Clinical Tuberculosis and Epidemiology Research Centre, National Research Institute of Tuberculosis and Lung Diseases (NRITLD), Shahid Beheshti University of Medical Sciences, Tehran, Iran; 6 Mycobacteriology Research Centre (MRC), National Research Institute of Tuberculosis and Lung Diseases (NRITLD), Shahid Beheshti University of Medical Sciences, Tehran, Iran; 7 Laboratory of Human Genetics of Infectious Diseases, Necker Branch, INSERM UMR 1163, Necker Hospital for Sick Children, University of Paris, Imagine Institute, 75015 Paris, EU France; 8 St. Giles Laboratory of Human Genetics of Infectious Diseases, Rockefeller Branch, The Rockefeller University, New York, NY USA; 9 Center for the Study of Primary Immunodeficiencies, Necker Hospital for Sick Children, AP-HP, Paris, EU France; 10 Howard Hughes Medical Institute, New York, NY USA

**Keywords:** Primary immunodeficiency, Mendelian susceptibility to mycobacterial disease, MSMD, Vasculitis, IL12RB

## Abstract

**Background:**

Mendelian susceptibility to mycobacterial disease (MSMD) is an inborn error of immunity, resulting in susceptibility to weakly virulent mycobacteria and other intramacrophagic pathogens. Rheumatologic manifestations and vasculitis are considered rare manifestations in MSMD patients.

**Case presentation:**

In this study, we reported a 20-year-old female who was presented with recurrent lymphadenitis following bacillus Calmette-Guérin (BCG) vaccination and a history of recurrent disseminated rash diagnosed as leukocytoclastic vasculitis (LCV). A slight reduction in lymphocyte subsets including CD4+, CD19+, and CD 16 + 56 T-cell count, as well as an elevation in immunoglobulins level (IgG, IgA, IgM, IgE), were observed in the patient. Whole exome sequencing revealed a homozygous Indel-frameshift mutation, c.527_528delCT (p. S176Cfs*12), at the exon 5 of the *IL12B* gene. She experienced symptom resolution after treatment with anti-mycobacterial agents and subcutaneous IFN-γ. We conducted a manual literature search for MSMD patients reported with vasculitis in PubMed, Web of Science, and Scopus databases. A total of 18 MSMD patients were found to be affected by a variety of vasculitis phenotypes mainly including LCV and Henoch-Schönlein purpura (HSP) with often skin involvement. Patients were all involved with vasculitis at the median age of 6.8 (2.6–7.7) years, nearly 6.1 years after the initial presentations. Sixteen patients (88.9%) had *IL12RB1* defects and concurrent *Salmonella* infection was reported in 15 (88.2%) patients.

**Conclusion:**

The lack of IL-12 and IL-23 signaling/activity/function and salmonella infection may be triggering factors for the development of leukocytoclastic vasculitis. IL12B or IL12RB1 deficiency and salmonellosis should be considered in MSMD patients with vasculitis.

## Background

Mendelian susceptibility to mycobacterial disease (MSMD) is a rare group of human inborn error of immunity (IEI) characterized by selective susceptibility to weakly virulent mycobacteria in otherwise healthy subjects, without overt immunological abnormalities [1]. Mycobacterial involvements may have a broad spectrum of clinical manifestations, from localized to disseminated, and acute to chronic infections. Although MSMD patients typically have normal resistance against other microbes, a number of viral infections, particularly herpes virus, bacterial, fungal, and parasitic infections have also been reported in patients with some underlying genetic defects [2]. Some MSMD patients are also particularly susceptible to *Salmonella spp* and develop a wide spectrum of clinical diseases, ranging from gastroenteritis to bacteremia [2–4]. Standard immunological tests for IEIs are generally normal in patients with MSMD [5]. Seventeen gene mutations are involved in MSMD (*IL12B*, *IL12RB1*, *IL12RB2, IL23R, JAK1, RORC, ISG15*, *TYK2*, *IRF8*, *SPPL2A, CYBB*, *IFNGR1*, *IFNGR2*, *STAT1*, *NEMO, TBX21,* and *ZNFX1*) [2, 5–7]. These genes are generally in the pathway of interferon gamma (IFN-γ), which is the macrophage-activating factor involved in anti-mycobacterium defense [8]. Leukocytoclastic vasculitis (LCV) is an immune complex mediated disease affecting small vessels of the skin and can be connected with drugs or be found as a component of other diseases, such as infections, connective tissue disorders, and malignancies [9]. LCV is considered a novel manifestation in MSMD patients and is mainly connected to microbial agents [10]. Biallelic mutations of *IL12RB1* are the most frequent genetic defect causing MSMD, and are found in about 60% of diagnosed patients [11]. In addition to the receptor for interleukin (IL)-12, IL-23 is also composed of IL-12Rβ1 and IL-23R subunits [12]. Therefore, patients with IL-12Rβ1 deficiency suffer from mycobacterial diseases due to IL12R and IL23R deficiencies, and chronic mucocutaneous candidiasis (CMC) due to impaired IL-23-dependent IL-17 production, while the etiology of salmonellosis like mycobacteriosis probably involves both IL12R and IL23R deficiencies given the relative rarity of salmonellosis in other MSMD etiologies [13]. Of note, IL-12 is involved in the generation of T helper (Th) 1 responses and production of IFN-γ [14], also, Th1 promotes immune responses against *Salmonella* species [15]. In this study, we identified a homozygous Indel frameshift mutation at the *IL12B* gene in a patient with recurrent lymphadenitis and leukocytoclastic vasculitis. We also reviewed the literature of MSMD cases presenting vasculitis.

## Methods

Patient information including demographic data, medical history, and physical examination, were collected by direct interviews and examining the patient’s clinical record based on national consensus on diagnosis and management guidelines for primary immunodeficiency [16]. Demographic data included age, gender, age at disease onset, age of diagnosis, and delay of diagnosis. The recorded laboratory data were: complete cell blood counts, T- and B-cells subsets (assessed using flow cytometry analysis), and serum levels of immunoglobulins and autoantibodies (assessed using nephelometry and enzyme-linked immunosorbent assay). Medical information was collected after obtaining written informed consent from the patient and his parents, following the principles of the ethics committee of the Shahid Beheshti University of Medical Sciences. Secondary causes of vasculitis were excluded by history taking, absence of renal and gastrointestinal involvement, and other drugs or disease-related causes. Clinical diagnosis of MSMD has been established according to the European Society for Immunodeficiencies criteria [17]. Genomic DNA extraction was performed using the whole peripheral blood sample. The whole-exome sequencing and confirmatory Sanger sequencing method were carried out according to a method published previously [18]. The literature search for reported MSMD patients was conducted in PubMed, Web of Science, and Scopus, applying the following keywords: “MSMD”, “Mendelian susceptibility to mycobacterial disease”, “Idiopathic infection caused by BCG or atypical mycobacteria”, “Mendelian susceptibility to atypical mycobacteria”, “Mendelian susceptibility to mycobacterial infections”, in combination with subsequent terminology: “vasculitis”, “Angiitis”, “hypersensitivity vasculitis”, “leukocytoclastic vasculitis”. Reference lists of all full-text articles and major reviews were manually searched for additional studies. The descriptive and comparative section was then developed on patients with MSMD patients.

## Results

### Case presentation

Our patient was a 20-year-old Baluch female, born to consanguineous parents from Sistan and Baluchestan. The patient has been studied previously along with other patients with MSMD [19]. She had a familial history of childhood death in her father’s cousins.

The proband was inoculated with bacillus Calmette-Guérin (BCG) at birth. She developed right axillary lymphadenitis at the age of 5 months following BCG vaccination and was treated with an anti-tuberculosis regimen for 3 months. At the age of 7 and 10 years old, she experienced recurrent cervical adenopathy and underwent lymph node excisional biopsy. The histopathology showed a reactive reaction with no sign of acid-fast bacilli. Later, she was hospitalized for general lymphadenopathy with abdominal lymph involvement at the age of 11 years. Further investigation was made through laparotomy and lymph node biopsy, which was accordant with reactive reaction and she was treated with antimycobacterial drugs for six months. She was referred to our hospital at the age of 12-year-old with complaints of cervical mass and weight loss. She went through full clinical, laboratory, and histological investigations. On physical examination, her weight and height growths were in the 10th percentile. She had bilateral cervical and inguinal lymphadenopathy. There were palpable purpuric rashes on her both legs resembling leukocytoclastic vasculitis.

Her biochemical markers such as serum hepatic enzymes, activated partial thromboplastin time, and prothrombin time were within normal ranges. No proteinuria, hematuria, or impaired renal function was detected. Blood analysis revealed normochromic normocytic anemia and a high erythrocyte sedimentation rate (125 mm/ first hour). Immunological investigation revealed a slight reduction in lymphocyte subsets including CD4+, CD19+, and CD 16 + 56 T-cell counts compared to age-adjusted-values. Other than hypergammaglobulinemia, the complement levels and neutrophil functions (Nitro blue Tetrazolium) were unremarkable. A purified protein derivative (PPD) skin test and microscopy of sputum samples for acid-fast bacilli, polymerase chain reaction (PCR), and culture for detecting *mycobacterium tuberculosis* were done and the results were negative (Table 1). Serologic tests ruled out other possible underlying causes of vasculitis. The biopsy of the skin lesion showed neutrophil infiltration in the dermal vessel walls compatible with leukocytoclastic vasculitis.

**Table 1:** Immunologic profile of the index patient

Parameters	Patient	Normal ranges
WBC × 10^3^ (cell/uL)	5.3	4.5–13.5
Hemoglobin (g/dL)	**7.7**	11.5–15.5
Monocytes (cell/uL)	0.5	0.2–0.8
Lymphocytes (cell/uL)	1.5	1.3–6.5
Neutrophils (cell/uL)	3.0	1.5–9.5
CD3^+^ T cells (% of lymphocytes)	63.9%	62.6–80.4 (%)
CD4^+^ T cells (% of T cells)	**31.4%**	32.6–51.5 (%)
CD8^+^ T cells (% of T cells)	20.8%	19.0–29.0 (%)
CD19^+^ (% of lymphocytes)	**6.9%**	11.9–21.0 (%)
CD16^+^ 56^+^ (% of lymphocytes)	12.3%	4.3–16.2 (%)
IgG (mg/dL)	**2700**	503–1719
IgA (mg/dL)	**425**	42–295
IgM (mg/dL)	**360**	41–255
IgE (IU/mL)	**100**	< 100
ESR (mm/first hour)	**125**	< 17
NBT (%)	99	> 80
C3 (IU/mL)	110	88–206
C4 (IU/mL)	38	13–75
CH50 (IU/mL)	80	42–95
T cell response to BCG	1.9	> 2.5
T cell response to PHA	3.1	≥3
T cell response to Candida	–	> 2.5
PPD	Negative	
ANA	Negative	
Anti-CCP	Negative	
p-ANCA	Negative	
c-ANCA	Negative	

*Ig* immunoglobulin, *WBC* white blood cell, *ESR* erythrocyte sedimentation rate, *NBT* Nitro blue tetrazolium, *ANA* antinuclear antibody, *Anti-CCP* anti-cyclic citrullinated peptide CH50; 50% hemolytic complement activity, *PHA* phytohemagglutinin, *BCG* Bacillus Calmette-Guérin, *PPD* purified protein derivative, *p-ANCA* Perinuclear anti-neutrophil cytoplasmic antibodies, *c-ANCA* Antineutrophil cytoplasmic antibodies

The histopathologic examination of the lymph node showed chronic lymphadenitis without acid-fast bacilli. At the time, she was treated with isoniazid (15 mg/kg/day), rifampin (10 mg/kg/day), ethambutol (20 mg/kg/day), ciprofloxacin (20 mg/kg/day), clarithromycin (15 mg/kg/day), and IFN-ɣ (50 μ/m^2^ every other day) for nine months and her clinical statusimproved after the treatment. However, she experienced multiple recurrence of LCV afterwards. During followup, dapsone and clofazimine were added to her regimen for 6 months. She experienced recurrence of lymphadenopathy, therefore, rifampin, isoniazid and ofloxacin (15 mg/kg/day) were discontinued and replaced by prothionamide (15–20 mg/kg/day), cycloserine (15–20 mg/kg/day), and later by levofloxacin (15 mg/kg/day), and fluvoxamine.

The patient relapsed at the age of 13 years and manifested right unilateral cervical lymph node enlargement. After cultivating the enlarged lymph node’s sample, a tuberculosis-like complex was detected but PCR sequencing of the sample was accordant to *M.Bovis*. Given her clinical and laboratory data, she underwent whole-exon sequencing for possible IFN-γ signaling pathway mutation. The genetic study identified a homozygous Indel-frameshift mutation, c.527_528delCT (p. S176Cfs*12), at the exon 5 of the *IL12B* gene and due to the type of her mutation, she was not a candidate for hematopoietic stem cell transplantation (HSCT).

In her lastest visit, despite the resolvation of her other symptoms with rifampin, isoniazid, levofloxacin, cycloserine, amikacin, ethambutol, and IFN-γ, she still suffered from LCV.

### Literature review

The literature search revealed 18 MSMD patients with vasculitis phenotype (9 females, 7 males, 2 unknown) with a median age of onset of 0.7 (0.3–5.0) year and diagnosis of 6.0 (3.0–10.0) year (Table 2). The most prevalent genetic defect in MSMD patients with vasculitis was *IL-12RB1* (16 of 18, 88.9%). Most of the patients were from Turkey 44.4%, followed by Iran and France (each 11.1%). Consanguineous marriage was reported in 76.9% (10 of 13 with available data) of patients and 42.9% (6 of 14) patients had a positive family history of immunodeficiency (most with similar symptoms). Fifteen patients were alive during the median follow-up of 12.0 (6.7–18.2) years (the life status of three patients was not available). About 79 % of patients (11 of 14) were vaccinated with BCG after birth and all of them developed BCG disease. However, only one patient presented with BCG-osis [29]. Patients were all reported to demonstrate vasculitis at the median age of 6.8 (2.6–7.7) years. Lymphadenopathy was the main first clinical presentation and involved all patients with available data. Furthermore, hepatomegaly and splenomegaly each appeared in 5 (41.7%) patients. Salmonellosis, as the most important concurrent infection in these patients, was detected in 88.2% (15 of 17). About half of patients (8 of 15) manifested candidiasis as well.

**Table 2:** An overview of the clinical and immunological findings in reported MSMD patients with vasculitis

NO.	Mutated geneion site	Mutation type	sex	Con.	FH.	AOO (Mo)	BCG vaccination	BCG disease	LAP location	Salmonellosis	Candidiasis	Vasculitis	Vasculitis age (Y)	Other manifestations	Immunologic abnormality	treatment	outcome	country	Ref.
P1	IL-12RB1	r.783 + 1G > A	M	+	+	60	NV	–	Cervical,mesentric,mandibular	Sd	–	Leukocytoclastic vasculitis	UN	Bacteriemia, Acinetobacter adenitis	–	AB	Alive	Turkey	[20]
P2	IL-12RB1	R173P	F	+	–	2	+	L	Axillary,submandibular,cervical,inguinal	Se,Sc	–	Leukocytoclastic vasculitis	7.0	Hepatosplenomagaly, mycobacterial abcess	–	AB,AM,INFG	Alive	Turkey	[21]
P3	IL-12RB1	557_563delins8	F	UN	UN	UN	+	L	UN	St,Se,Stm	+	Vasculitis	UN	UN	UN	UN	Alive	Turkey	[22]
P4	IL-12RB1	783 + 1G > A	F	UN	+	UN	NV	–	UN	SD,SB	+	Leukocytoclastic vasculitis	UN	UN	UN	UN	Alive	Turkey	[22]
P5	IL-12RB1	783 + 1G > A	F	+	–	8	+	L	Axillary,submandibular	Se	+	Leukocytoclastic vasculitis	1.83	bacteriemia	Hypogammaglobulinemia	AB	Alive	Turkey	[10]
P6	IL-12RB1	1791 + 2 T > G	M	–	–	4	+	L	Axillary,cervical,peripheral	Se	–	Leukocytoclastic vasculitis	0.58	Hepatosplenomagaly, Bacteriemia	–	AB,AM	Alive	Tunisia	[23]
P7	IL-12RB1	UN	M	+	+	84	UN	UN	Abdominal	St	–	Leukocytoclastic vasculitis	7	Splenomegaly, bacteriemia *K. kristinae*,skin rash,	Positive ANA,Coombs HIgA,HIgG Low CD4+, high CD8+	AB	Alive	Israel	[24]
P8	IL-12RB1	c.21G > A	M	–	–	6	+	L	Axillary	+	–	Leukocytoclastic vasculitis	5	Ankle arthritis,pytiriasis	Positive RF, Hypogammaglobulinemia	AB,AM	Alive	Brazil	[25]
P9	IL-12RB1	c.1561C > T	UN	UN	UN	UN	UN	UN	UN	+	+	Leukocytoclastic vasculitis	UN	Disseminated histoplasmosis	UN	UN	UN	France	[11]
P10	IL-12RB1	c.523C > T	F	–	–	12	+	L	Axillary	St,Stm	+	Leukocytoclastic vasculitis	UN	mycobacterial abcess	Low CD3+, Hypogammaglobulinemia	UN	Alive	Turkey	[26]
P11	IL-12RB1	c.261C > A	M	+	–	24	UN	UN	Axillary, cervical, mesenteric	St	UN	Urticarial vasculitis	UN	Typhoid fever	Low CD4+, Low CD19+, HIgA	UN	Alive	Turkey	[26]
P12	IL-12RB1	c.962C > A	M	+	+	1	+	L	UN	St	+	Leukocytoclastic vasculitis	UN	Hepatomegaly mycobacterial osteomyelitis, prolonged diarrhea,bacteriemia,IBD like lesions,AIHA	Positive ANA,Coombs	AB+AM+prednisolone	Alive	India	[3]
P13	IL-12RB1	c.517C > T	M	+	+	UN	+	+	Bilateral	+	–	Henoc-Schönlein purpura	UN	Hepatosplenomagaly, liver abscess, osteomyelitis,seizure,meningitis due to TB	HIgG, hypo IgM	INFG	UN	Iran (Turk)	[27]
P14	IL-12RB1	c.1791 + 2 T > G	F	+	UN	UN	+	+	Bilateral	–	+	Henoch-Schönlein purpura, Vasculitis	UN	Hepatosplenomagaly, severe diarrhea	HIgG, HIgA, HIgE	UN	UN	Iran (Fars)	[27]
P15	IL-12RB1	UN	UN	UN	UN	UN	+	L	+	Se	UN	Vasculitis	8	UN	Positive RF, Hypogammaglobulinemia	AB,INFG, Glucocorticoids	Alive	Russia	[28]
P16	IL-12RB1	c.783 + 1G > A	F	+	+	9	NV	–	+	+	+	Leukocytoclastic vasculitis	UN	UN	UN	UN	Alive	Turkey	[28]
P17	IFNGR2	R114C	F	+	–	UN	+	D	Cervical	–	–	Disseminated skin vasculitis	16	Cellulitis, *M. abscessus* infection	UN	AB,AM,INFG	Alive	France	[29]
P18	IFNGR1	818del4	F	UN	+	78	UN	UN	Cervical,inguinal	–	–	Henoc-Schönlein purpura	6.7	Lung abcess, liver abcess with *E. faecium*	UN	AB,AM,INFG	Alive	Norway	[30]

*UN* Unknown, *M* Male, *F* Female, *Con* Consanguinity, *FH* Family history, *AOO* Age of onset, *Mo* Month, *BCG* Bacille Calmette-Guerin, *D* disseminated, *L* localized, *NV* Not vaccinated, *LAP* Lymphadenopathy, *Stm* S. typhimurium, *Se* S. enteritidis, *SB* S. group B, *SD* S. group D, *St* S. typhi, *ANA* Anti-nuclear antibody, *AIHA* Autoimmune hemolytic anemia, *RF* Reumatoid factor, *HIg* High immunoglobin level, *AB* Anti-biotic, *AM* Anti-mycobacterial, *INFG* Interferon gamma, *INFGR2* Interferon gamma receptor 2, *IL-12RB1* Interleukin 12 receptor beta 1

The normal immunological parameters were the most reported results in MSMD patients with vasculitis. Moreover, high IgG, high IgA, and high IgM were detected in 72.7% (8 of 11), 72.7% (8 of 11), and 45.5% (5 of 11) of the patients. Anti-mycobacterial agents were the most supportive treatments and 5 patients underwent IFN-γ therapy. All patients treated with IFN-γ, are alive and achieved resolution of major symptoms.

## Discussion

MSMD is an early-onset primary immunodeficiency that mainly presents mycobacterial lymphadenitis [5]. Here, we described a 20-year-old Iranian female presented with a homozygous mutation, c.527_528delCT (p. S176Cfs*12) of the *IL12B* gene. She suffered from recurrent lymphadenitis since infancy and later manifested LCV. As opposed to most patients with LCV [4, 10, 23], *Salmonella* species were not isolated from our patient and she did not experience candidiasis either. She is the second patient with IL-12B/RB deficiency manifested with LCV without *Salmonella* infection. Most of the reported MSMD patients with vasculitis (*n* = 16, 88.9%) had *IL12RB1* defects, and concurrent *Salmonella* infection was reported in 15 (88.2%) patients as IL-12 pathway is involved during *Salmonella* infection [15]. Moreover, LCV in MSMD patients predominantly presented with palpable purpuric rashes located on lower extrimities and were compeletly resolved following the use of 3rd generation cephalosporins as part of the anti-tuberculosis regimen, ciprofloxacin alone or in combination with INF- γ [10, 21, 23, 24], further strengthening the possible role of salmonella infection in the development of LCV. Subacute salmonellosis or lack of appropriate follow-up might be the reason of not detecting Salmonella species in some cases. Therefore, the lack of IL-12 and IL-23 immunity and salmonella infection may be triggering factors for the development of vasculitis. However, our patient as an outlier, experienced recurrent episodes of LCV that did not relieve even after the resolvation of her other symptoms or treating with anti-tuberculosis and anti-salmonella drugs.

Laboratory analysis detected positive RF and a slight decrease in CD4+, CD19+, and CD 16 + 56 T-cell count. Döffinger et al. have also reported an *IFNGR2*-deficient patient presented with vasculitis but no sign of *Salmonella* or *Candida* infections [29]. Similar to Kutukculer N et al., our patient did not achieve symptom resolution before the initiation of IFN-γ therapy [21].

Vasculitis is a phenomenon that could be associated with infection, autoimmune and inflammatory conditions including medications. Various infectious agents can cause vasculitis directly or clinically mimicking primary vasculitis [31]. Hypersensitivity vasculitis is a rare manifestation occurring during an atypical mycobacterial infection in a healthy subject [32, 33]. Furthermore, there are a few exceptional MSMD-deficient patients that presented with LCV, especially patients with *IL-12Rβ1* deficiency [3, 26, 27]. Our review of 18 MSMD cases with vasculitis revealed that this complication appears in middle childhood in patients predominantly originated from Turkey. Since Th1 is involved in ANCA-associated vasculitis and a majority of patients with MSMD developed Henoc-Schönlein purpura or LCV, the etiology of vasculitis in MSMD might not be Th1-mediated [34]. Noteworthy, most patients were positive for *Salmonella* species, suggesting an association for vasculitis and salmonellosis in the cases. On the other hand, it is possible that subclinical *Salmonella* infections or lack of appropriate follow-up in patients are the underlying reasons for negative *Salmonella* isolation in some cases. Candidiasis was also common among the reviewed patients, however, most of our reported patients had *IL-12RB* deficiency and this mutation is previously known to positively correlate with candidiasis [5]. As expected, laboratory data were predominantly normal including lymphocyte subsets, however, most patients were detected with hyperimmunoglobulinemia. BCG vaccination seems to be an important factor as all vaccinated patients developed BCG disease. Therefore, investigating the family history of abnormal post-vaccination complications or atypical mycobacterial infections before BCG administration would benefit MSMD patients with susceptibility to infection with weakly virulent mycobacteria.

## Conclusion

In conclusion, cutaneous vasculitis in MSMD patients could be pathognomic of an underlying IL12B or IL12Rβ1 deficiency, particularly in those complicated with *Salmonella* infections. Furthermore, although vasculitis is a relatively late symptom, clinicians should be aware of possible underlying causes of cutaneous vasculitis in children presenting with unusual recurrent *Salmonella* and weakly virulent mycobacteria infections and investigate for possible IFN-γ pathway mutations.

## Data Availability

All data generated or analysed during this study are included in this published article.

## Abbreviations

MSMD: Mendelian susceptibility to mycobacterial disease
BCG: Bacillus Calmette-Guérin
LCV: Leukocytoclastic vasculitis
HSP: Henoch-Schönlein purpura
IEI: Inborn error of immunity
PPD: Purified protein derivative
PCR: Polymerase chain reaction
HSCT: Hematopoietic stem cell transplantation
